# Exploring the Construct of Relational Values: An Empirical Approach

**DOI:** 10.3389/fpsyg.2020.00209

**Published:** 2020-03-13

**Authors:** Matthias Winfried Kleespies, Paul Wilhelm Dierkes

**Affiliations:** Department of Biology, Bioscience Education and Zoo Biology, Goethe University Frankfurt, Frankfurt, Germany

**Keywords:** relational values, connectedness to nature, connectedness to nature scale, scale validation, environmental behavior, two factor model of environmental values model

## Abstract

In recent environmental research, relational values (RVs) have emerged as a new group of values to explain environmental behavior. Although this new concept is attracting attention, empirical studies on the subject are still rare. On this basis, we have conducted three studies to analyze an existing measurement tool for RVs and compared the construct with the concept of connection to nature. In study 1, we confirmed convergent and discriminant validity of the RV scale by comparing it with the Two Factor Model of Environmental Values (2-MEV) model using a sample of *n* = 350 university students. Additionally, study 1 verified reliability using test–retest reliability on three different groups of students (*n*_1_ = 53; *n*_2_ = 37; *n*_3_ = 48). In study 2, principal component analyses were performed to examine the structure of RVs and to compare it to the concept of connection to nature by reusing the sample 350 university students from study 1. The results show that RVs and connection to nature are not fundamentally distinct constructs, but overlap. However, if the structure of the RV measurement is forced to a single factor, no perfect fit is found, making a multidimensional solution more likely. A third study was conducted to review the results from study 2 using confirmatory factor analysis on a new sample of 878 university and high school students. Study 3 confirmed RVs as a multidimensional construct with three factors: care, community, and connection. It also proved the overlap of the connection to nature and RV concepts to some extent.

## Introduction

Why should nature be preserved and protected? This question is often answered from an instrumental or an intrinsic position (Tallis and Lubchenco, 2014). The fundamental postulate of the intrinsic perspective is that biological diversity has a value of its own, regardless of the potential use or benefit for humans. Species have value because of their pure existence, and this value is seen as inviolable (Soulé, 1985; Sandler, 2012). In terms of the instrumental point of view, the focus is on ecosystem services for the protection of nature. Instrumentalists argue that this beneficial approach is more effective than an intrinsic value of nature (Reid et al., 2006). Furthermore, protection of nature for its own good is considered as outdated and impractical (Soulé, 2013). A weakness of the instrumental view is the existence of natural things that have little or no value to humans. For this reason, intrinsic values should not be disregarded. However, instrumental arguments are more often effective for the general public and should therefore be used in contexts where conservation is crucial (Tallis and Lubchenco, 2014).

As a rule, human decisions are not only based on intrinsic values or the usability of nature. Hence, a third group of values explaining environmental behavior has recently gained attention: relational values (RVs). This original philosophical term includes human beliefs on what is the right and appropriate way to deal with nature. RVs reflect the responsibility and relationship humans have toward nature and the place where they live. In addition, RVs give rise to questions regarding how to deal with nature and the land to live a good and meaningful life. But RVs include not only the relationships of humans with nature and the responsibility associated with them but also the relationships and decisions between people involving nature. In this way, RVs are involving the human collective as well as the individual. Natural objects do not contain RV *per se*, but RVs are the results of the relationship or commitment to them (Chan et al., 2016).

RVs are not an end in itself but provide important factors for environmental conservation, sustainability, and living a meaningful life (Himes and Muraca, 2018). The practical use of RVs can be explained by the example of protected areas. Without RVs, protected areas have to justify their existence in an instrumental or intrinsic way. This means that those areas either have to provide some kind of benefit for humans, most of the time valued in ecosystem services, or are valued “just” for their own sake. RVs add a third option, namely, to appreciate nature because of the people’s relationship to it and the responsibility toward other people (De Vos et al., 2018).

In the last years, a number of articles on the topic of RVs have been published (e.g. Chan et al., 2016; Arias-Arévalo et al., 2017; Chapman, 2017; Klain et al., 2017; Chan et al., 2018) that helped to achieve a better understanding of the RV concept and displayed the strength and relevance of RVs to a wide range of scientists in different research fields. For example, Ishihara (2018) made a noteworthy contribution to the topic of RVs from the sociology perspective. She integrated RVs in the concept of Pierre Bourdieu’s habitus. RVs can be seen as a part of this habitus that is expressed by culture and the social group an individual is part of. Beyond that, RVs also have a great influence from the social perspective, and the violation of these values has a strong impact on the affected community. If, for example, an important place for the community is destroyed, it could also lead to the loss of relationships between humans and nature (Grubert, 2018). This is a critical difference to the instrumental values. When a habitat is destroyed, from an instrumental view, it can be relocated to another place and still provide the same ecosystem services than before. For RVs, it is different. The bond and emotions connected to a place cannot be relocated (Grubert, 2018). This context clearly demonstrates the importance of RVs, although they are criticized for not being a useful categorization by some researchers (Maier and Feest, 2016).

In addition, the connection of RVs and the conservation of biodiversity on agricultural land has to be mentioned. Large tracks of land, locally and globally, are used for agricultural purposes. The cultural RVs help to sustain biodiversity and particularly the species diversity in these areas. When politics focus on instrumental values and economically orientated decisions, less actions in conservation will be taken (Allen et al., 2018). Hence, RVs may be a crucial tool to influence politics to reach a more sustainable use of nature by addressing a broad spectrum of people: stakeholders, scientists, local community, managers, and politicians (Stenseke, 2018).

Even if RVs get a lot of attention in review articles and qualitative analysis, there is a lack of quantitative research on this topic. Britto dos Santos and Gould (2018) argue that environmental education has already approached RVs without defining them as such. For the authors, connectedness, care, community, identity, kinship, responsibility, and stewardship are all part of RVs.

In psychology, there are various approaches that try to integrate the concept of values into a theoretical framework. Values are defined as ideas or beliefs regarding desirable goals or behavior. They go beyond certain situations, help to evaluate behavior or events, and can be ranked according to their importance (Schwartz and Bilsky, 1987). The concept of values clearly differs from the concept of attitudes, which expresses a positive or negative tendency toward an entity (Eagly and Chaiken, 1993). The fundamental difference between values and attitudes is the generality and the hierarchical order of importance of values (Schwartz, 1992). Values are broader than attitudes and therefore serve as an organizational system for attitudes and beliefs (Schultz et al., 2004). Attitudes have a certain influence on behavior, even if they cannot be translated one-to-one into behavior (Inglehart, 1997; Hitlin and Piliavin, 2004). Beliefs are personal conceptions, which assign a truth content to a fact. In contrast to values, beliefs are not guiding principles in life but allow a quick personal assessment of the plausibility of situations and contexts (Schwartz, 2012). Values and beliefs are important basic components of personal identity (Hitlin, 2003) and thus as a way to organize information about oneself (Clayton, 2003).

The construct of RVs seems to overlap with existing validated environmental psychological constructs, for example, with the theory of basic human values developed by Schwartz (1992). The author identified 10 universal values that display the essential basic needs of humans. These 10 values can be organized in four higher ordered factors: openness to change, self-transcendence, conservation, and self-enhancement. Especially the factor self-transcendence that includes benevolence and universalism seems to overlap with RVs. The goal of benevolence is to preserve and enhance the welfare of the community a person is in contact with, while universalism aims to understand, appreciate, tolerate, and protect the welfare of nature and all humans (Schwartz, 2012). The combination of nature and society is a factor that occurs in both concepts.

In addition, the concept of Stern and Dietz (1994) has some similarities with RVs. They explain environmental concerns through three kinds of values: egoistic, altruistic, and biospheric values. Egoistic values focus on parts of the environment that effect a person directly. If environmental changes adversely affect people, the person concerned should act in a more environmentally friendly way to avoid the consequences. The altruistic values describe the moral responsibility not to harm other people, while the biospheric values account the cost and benefits for an ecosystem or the biosphere (Stern and Dietz, 1994). The three values influence the personal beliefs and norms and in this way cause proenvironmental behavior (Stern, 2000). A proenvironmental decision can be motivated by all three kinds of values. For example, buying a smaller and energy efficient car can be motivated because it is cheaper (egoistic values), because it produces smaller amounts of toxic gases that could endanger the health of other people (altruistic values), or because it produces less carbon dioxide (CO_2_) to protect the environment (biosphere values) (De Groot and Steg, 2010). Once again, RVs overlap with a known concept of environmental psychology. Both constructs involve a personal relation to nature but also the decisions and relationships that involve other people.

Another important concept that has to be mentioned in this context is connection to nature. Connection to nature is a well-known construct in environmental education research and environmental psychology and shows a certain resemblance to RVs.

Although connection to nature receives so much attention, there is no clear and universal definition. Some authors particularly emphasize the emotional focus of the concept (e.g. Mayer and Frantz, 2004; Nisbet et al., 2009), while others consider, for example, the role of nature in personal identity (Clayton, 2003). For our research, we would like to draw on the conceptual statements of Schultz (2002), who describes the connection to nature as the belief in how strongly a person sees himself or herself as part of the natural environment. Schultz (2002, p. 67) defined connection with nature as “*[*…*] the extent to which an individual includes nature within his/her [.] self*.” He describes his concept of inclusion with nature with three core dimensions. The cognitive component describes the feeling to be integrated in nature, and the affective component includes the care for nature, place, and animals. The third component is behavioral. When people are connected to and care for nature, they are more motivated to act in the interest of nature (Schultz, 2002).

Environmental psychologists developed a number of measurement tools for natural connectedness: These include the Nature Relatedness Scale by Nisbet et al. (2009), the Environmental Identity (EID) Scale by Clayton (2003), the Connectedness to Nature Scale (CNS) by Mayer and Frantz (2004), the Inclusion of Nature in Self (INS) Scale by Schultz (2002), and the Implicit Association with Nature Scale by Schultz et al. (2004). Most of the mentioned concepts show intercorrelation and can therefore be considered as a single construct (Tam, 2013). Some authors have demonstrated that the concept of connectedness to nature can be taken more widely and is related to environmental values or identity (Brügger et al., 2011; Olivos and Aragonés, 2011). On the basis of the abovementioned research, it can be concluded that RVs as well as the concept of connection to nature contain the personal relationship to nature. In addition, the connection and care of places have important roles in both constructs.

Connection to nature has gained a lot of attention in environmental psychology as well as in interdisciplinary research. The different measurement tools for connection to nature are widely used to evaluate environmental education programs and explain proenvironmental behavior (Kals et al., 1999; Frantz et al., 2005; Kaiser et al., 2008). In most modern western societies, adults and children spend more time indoors and less time in natural environments. This development obviously has a negative effect on the connectedness to nature. Children who are spending more time playing indoors, watching TV, or playing video games have a lower implicit connectedness to nature (Bruni and Schultz, 2010). Children develop connection to nature through positive nature experiences and the time spent in nature as a child is an important predictor for the time spent outside later in life (Rosa et al., 2018). On the other hand, time spent outdoors leads to increased connection with nature (Schultz and Tabanico, 2007; Kaiser et al., 2008; Andrejewski et al., 2011; Dornhoff et al., 2019).

Why is the decrease of affinity to nature a problem? Various studies are dealing intensively with the relationship between connection to nature and proenvironmental behavior. People with a stronger connection to nature show more protective behavior compared to people with lower connection (e.g. Kals et al., 1999; Frantz et al., 2005). Furthermore, a higher connection supports environmental attitudes and more motivation to preserve nature (Wells and Lekies, 2006; Kaiser et al., 2008). In summary, people with a closer connection to nature tend to be more environmentally friendly (Clayton, 2003) and show an increase in proenvironmental behaviors (Geng et al., 2015). Additionally, a higher connection to nature leads to higher vitality, lower mental distress, and higher psychological well-being (Cervinka et al., 2011; White et al., 2013; Capaldi et al., 2014).

While there are many studies examining connection to nature and the effect on proenvironmental behavior with empirical data by now, recently, only two studies with a quantitative approach to examine RVs have been published (Arias-Arévalo et al., 2017; Klain et al., 2017). One reason could be the lack of a validated measurement instrument for RVs. An extension to more quantitative research on RVs could provide solid empirical evidence and confirm the relevance of RVs in environmental research and conservation. Additionally, global aspects of RVs could be identified by checking for commonalities or differences in different cultures or countries to promote conversation and collaboration between these groups. Finally, statistically, representative surveys can reflect the public view and therefore help politics to make environmentally beneficial decisions (Schultz and Martin-Ortega, 2018).

A fundamental problem in the empirical research context on RVs is the lack of an established test instrument that has been checked for its test quality criteria. In study 1, we want to close this gap and verify the validity and reliability of an RV measurement tool developed and used by Klain et al. (2017). This necessary step is important for the development and establishment of a reliable and valid measuring instrument with which the construct can be examined in more detail. To confirm validity and reliability, the RV instrument was compared to the Two Factor Model of Environmental Values (2-MEV) model. The 2-MEV model was chosen because it is of great importance in environmental research, and its quality and structure have often been successfully confirmed (e.g. Milfont and Duckitt, 2004; Johnson and Manoli, 2010; Boeve-de Pauw and Van Petegem, 2011). Since there is also a gap in empirical research on the structure of the RVs, we used the RV measurement instrument (after both discriminant and convergent validity and the test–retest reliability were confirmed in study 1) to empirically investigate the structure of the RV construct (studies 2 and 3). In the first step (study 2), a structure-discovering method [principal component analysis (PCA)] was selected to obtain a basic overview of the factor structure of RVs. In order to further refine the results of study 2, the structure was then examined in study 3 with a new sample using structure-checking methods [confirmatory factor analysis (CFAs)]. Since connection to nature is very important in the context of environmental education and environmental psychology, and since we found some theoretical similarities between the concept and RVs, we additionally investigated the structural relationship between the RV items and connection to nature in studies 2 and 3. For this purpose, items of a well-known and established measurement instrument for connection to nature, the CNS by Mayer and Frantz (2004), were used.

## Study 1

In study 1, we tested the measurement tool used by Klain et al. (2017) for its convergent and discriminant validity. For this purpose, we compared the RV measurement with the well-known 2-MEV model using a sample of 350 university students. In a second step, we examined the reliability of the RV measurement scale using test–retest reliability on three different samples: a group of university students and two groups of high school students.

### Methods

#### Measurement of Relational Values and the Two Factor Model of Environmental Values Scale

Since RVs have only recently received more attention in empirical research, there are no established and repeatedly used measurement instruments available. Nevertheless, there have been several attempts to measure RVs. Chapman (2017) conducted interviews with 22 farmers and land managers, wherein they identified different values, including RVs. This approach is probably effective, although it requires a lot of time and considerable effort. Arias-Arévalo et al. (2017) chose an open-ended questionnaire to measure instrumental, intrinsic, and relational values. Using the same approach as Chapman (2017), they assigned values to the statements, and data analysis revealed that intrinsic and relational values outweighed instrumental values. Furthermore, Uehara et al. (2018) developed seven relational statements based on the seven definitions for RVs by Chan et al. (2016). Participants had to rate the statements on a five-point Likert scale from strongly disagree (1) to strongly agree (5).

Our study is based on a questionnaire developed by Klain et al. (2017) who modified seven value statements derived from studies about cultural ecosystem services (Appendix 1). Each of the items used emphasizes a different focus: community, health, identity, kinship, responsibility, wild places, and environmental impacts.

In terms of content, the seven statements fit well with the definition of RVs. The community item covers the aspect of RVs that sees place and nature as a tool to connect people to a community. The health item describes nature as an aid to increase health and well-being of a person and other people. This aspect is an important part of RVs. The items identity and kinship reflect the personal connection to plants, animals, and land as a part of the personal identity. The remaining three items (responsibility, wild places, and environmental impacts) include the stewardship and care for nature, special places, and other people. This relationship shows that the seven items applied well represent the construct of RVs.

The item responsibility had to be slightly revised because the questions were originally used to consult farmers and tourists. Instead of “How I manage the land […],” we asked, “How we manage the land […].” We translated the items to German and, similar to the original study, our participants had to rate the statements on a five-point Likert scale.

##### Two factor model of environmental values scale

The 2-MEV Scale is a well-known measurement tool for ecological values developed by Bogner and Wisemann (2006). The model consists of two higher factors: preservation and utilization. Preservation represents the preference to conserve and protect nature, and utilization represents the use or exploitation of nature for benefits. The scale is used regularly, and its validity and structure are proven many times (e.g. Milfont and Duckitt, 2004; Johnson and Manoli, 2010; Boeve-de Pauw and Van Petegem, 2011). In recent research, Bogner (2018) extended the scale with a third factor: appreciation of nature.

For our study, we selected the five highest loading items on preservation as well as on utilization from Bogner and Wisemann (2006). Furthermore, we added the five highest loading appreciation items from Bogner (2018).

#### Procedure and Participants

##### Convergent and discriminant validity

To test for convergent and discriminant validity, 350 biology students (69.4% female, 27.7% male, 2.9% no answer) were surveyed. More than 90% of the study participants were aged between 18 and 26 years. The participation in our survey was voluntary, and all respondents were of full age. A majority of the students (*n* = 271) were enrolled in the course “Diversität der Organismen & Lebensräume” (Diversity of Organisms & Habitats) at Goethe-University Frankfurt. This basic course is usually taken in the second semester of the Bachelor of Science (Biology) and Teacher Training in Biology programs. The questionnaires were handed out at the beginning of the courses, and the students decided for themselves if or when they wanted to fill in the survey. To prevent coercion, the students were asked to leave the questionnaire on their table after the course, so it was possible to give back empty questionnaires anonymously. The other 79 participants answered the questionnaire during Bioscience Education seminars in the same month (July 2018). We received 350 out of 450 questionnaires, which corresponds to a response rate of 77.78%.

##### Test–retest reliability

For verifying test–retest reliability, we selected new groups that have not previously participated in the validity test. The reason for this is a possible influencing of the results if the participants had seen and completed the questionnaire before. The recommended sample size for testing test–retest reliability is at least 100 (Kline, 1999). To prove test–retest reliability, the measurement tool was tested over three different time intervals. We asked university students who participated in the course Human Biology (M_age_ = 24.15 +2.44) to complete the questionnaire at the beginning and at the end of the winter semester 2018/2019 3 months later (mid November 2018 and mid January 2019). From the initial 73 students, 53 completed both questionnaires. The second group consists of 45 pupils from a local school who participated in a program called Goethe Biolab week (M_age_ = 16.17 +0.877). The second questionnaire was conducted 5 days after the first one by 37 pupils. This part of the survey took place in April 2019. Forty-eight pupils participated in the last group (M_age_ = 16.80 +0.707), visiting the Opel Zoo in Kronberg (Germany) in March 2019. All students of this group attended to both questionnaires before and after the zoo visit, 2 h later. For their participation, the students got free entrance to the zoo and a guided tour. If students did not complete the questionnaire, they did not suffer any disadvantages. The survey participation was voluntary, and participants under the legal age had to bring a signed letter of agreement by their parents. Privacy policy has been respected.

#### Analysis

All statistical analyses were executed using IBM SPSS 24. To assess the discriminant and convergent validity of the RV scale and the Pearson correlation between RVs and the appreciation, the preservation as well as the utilization items were calculated.

To determine the test–retest reliability, the Pearson correlation coefficient between the two measurement points of the three groups was calculated.

### Results

The Pearson correlation between the seven RV items and the five appreciation items was medium (*r* = 0.469; *p* < 0.001), between RV and the preservation items high (*r* = 0.553; *p* < 0.001), and between RV and the utilization items negative (*r* = −0.284; *p* < 0.001).

For the 3-month test group, the value of the correlation coefficient was *r* = 0.837 (*p* < 0.001; M_1_ = 3.64 +0.790; M_2_ = 3.61 +0.696), *r* = 0.720 (*p* < 0.001, M_1_ = 3.75 +0.664; M_2_ = 3.79 +0.648) for the 5-day group and *r* = 0.756 (*p* < 0.001; M_1_ = 3.61 +0.680; M_2_ = 3.73 +0.750) for the 2-h group.

### Discussion

#### Convergent and Discriminant Validity

Convergent and discriminant validities are important parts of construct validity. A measurement tool is considered as convergent if it is related to other measurement tools that measure the same or a similar construct. In contrast, a measurement tool is classified as discriminant when it is unrelated to measurement tools measuring distinct constructs (Campbell and Fiske, 1959). A common approach to evaluate both kinds of validity is to use Pearson correlation (Lehmann, 1988). For convergent validity, a correlation under *r* = 0.5 should be avoided (Carlson and Herdman, 2012).

In our study, we expected a positive correlation between RV and the appreciation items and between RV and the preservation items because all three are potential motivators for people to conserve nature (Wiseman and Bogner, 2003; Van den Born et al., 2017; Bogner, 2018). The calculated Pearson correlation coefficient between RVs and appreciation for nature almost showed a high effect (*r* = 0.469). For RVs and preservation, the correlation was high (*r* = 0.553). These results add evidence for convergent validity of the RV construct because preservation, appreciation, and RVs reflect a positive attitude toward nature. Comparable studies for other measurement tools reported similar correlations to verify convergent validity (Mayer and Frantz, 2004; Perkins, 2010; Olivos et al., 2011; Pasca et al., 2017).

In comparison, for discriminant validity, the correlation with divergent measuring instruments should be noticeably lower than the correlation with convergent measuring instruments (Hubley, 2014). We expected a divergence between RVs and utilization as the utilization items describe nature as an object that can be used by humans. This assumption was underlined with the obtained correlation (*r* = −0.284), adding evidence for discriminant and therefore for construct validity.

#### Test–Retest Reliability

To test the consistency of a measurement, reliability should be determined. In addition to the α-coefficient, the test–retest method is a common approach to verify reliability. For the test–retest, the same questionnaire is applied to the same test group on different occasions, and results are compared by correlation (McIntire and Miller, 2007). A correlation of *r* ≥ 0.7 represents an acceptable reliability (Domino and Domino, 2006).

There are several factors influencing the result of the test–retest reliability. For example, the time gap between the testing and the sample size. Test–retest reliability is unsuitable for testing knowledge or skills because the first test is a practice for the second one, and participants could remember the answers (Domino and Domino, 2006). RVs are a part of human attitudes toward nature, and as part of the habitus (Ishihara, 2018), they should not change over a short period of time. The results revealed a sufficient correlation for all tested groups adding further evidence for reliability of the measurement tool. The 3-month group reached the highest value, while the 5-day and the 2-h groups scored slightly lower but still over the cutoff value of *r* ≥ 0.7.

## Study 2

In study 2, we executed a series of PCAs on the same sample of 350 university students used in study 1 (Section “Convergent and Discriminant Validity”) to explore the fundamental structure of the concept of RVs. In this context, we compared an established CNS with the measurement of RVs to explore the overlap of these constructs. To this end, we used a shortened version of CNS.

### Methods

In a first step, a PCA with the seven CNS items was conducted to confirm the single-factor structure of the shortened CNS version for our test group. In a second step, a PCA was used to explore the structure of the seven RV items. Finally, a third PCA was applied to examine the connection between CNS and RVs.

#### Measurement

##### Connectedness to nature scale

Connectedness to nature scale was developed by Mayer and Frantz (2004) to measure the connectedness to nature. In contrast to other measuring instruments, such as the New Ecological Paradigm (NEP) (Dunlap et al., 2000), CNS measures the affective experience rather than the cognitive beliefs. Since CNS is a 14-item scale, it is possible to assess it for reliability [in contrast to INS Scale by Schultz (2002)]. Perrin and Benassi (2009) criticize the CNS construct because their study shows that CNS does not measure emotional connection to nature. They argue that Mayer and Frantz used words that do not imply emotional connectedness (e.g. I fell [.]). Nevertheless, Perrin and Benassi (2009) agree that CNS is a suitable tool to measure beliefs regarding connection to nature. The scale has often been used in recent studies, and its reliability has been repeatedly confirmed (e.g. Mayer et al., 2009; Gosling and Williams, 2010; Cervinka et al., 2011; Olivos et al., 2011; Zhang et al., 2014; Navarro et al., 2017).

For our study, we selected seven of the 14 items with the highest factor loading, provided by Mayer and Frantz (2004), to keep the questionnaire compact. Since Mayer and Frantz (2004) carried out five surveys in their study, the factor loadings were averaged based on the number of participants. Validity, reliability, and correct measurement of connectedness to nature were proven for a reduced CNS (Pasca et al., 2017).

#### Participants

For study 2, the data set of the 350 university students from study 1 was reused. The sample size was adequate according to Comrey and Lee (1992), who suggest at least *n* = 300 for factor analysis.

#### Analysis

All statistical analyses were executed using IBM SPSS 24. To investigate the relationship between CNS and RVs, a series of PCAs were performed. The Bartlett test and the Kaiser–Meyer–Olkin (KMO) test were applied.

### Results

A PCA with oblique rotation was conducted to confirm the single-factor structure of the reduced CNS. The number of factors was set at one, as proposed by the developers of the scale (Mayer and Frantz, 2004) and other authors (e.g. Olivos et al., 2011). The first factor accounted for 57.7% of the variance after the lowest loading item (>0.3) was removed (Table 1).

**TABLE 1:** Result of the principal component analysis with oblique rotation for shortened Connectedness to Nature Scale (CNS).

		**Factor loading**
CNS_6	I often feel part of the web of life.	0.827
CNS_5	Like a tree can be part of a forest, I feel embedded within the broader natural world.	0.805
CNS_2	I often feel a sense of oneness with the natural world around me.	0.794
CNS_4	I think of the natural world as a community to which I belong.	0.768
CNS_1	When I think of my life, I imagine myself to be part of a larger cyclical process of living.	0.680
CNS_7	I feel as though I belong to the Earth as equally as it belongs to me.	0.666
α = 0.849		
		

The six remaining items had factor loadings between 0.666 and 0.827. The KMO test approved the sampling adequacy (KMO = 0.869). Furthermore, the Bartlett test was highly significant; therefore, the conditions for factor analysis were fulfilled. To verify the reliability, Cronbach’s alpha was determined (α = 0.849).

**FIGURE 1:**
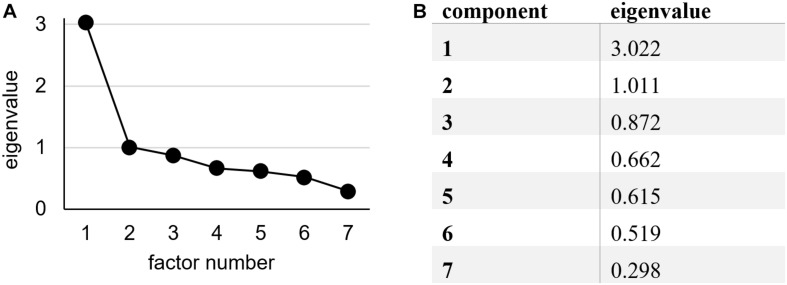
**(A)** Scree plot and **(B)** eigenvalues of the seven factors of the principal component analysis (PCA) with the seven relational value (RV) items.

The second PCA was used to examine the internal coherence of the seven RV items as a single dimension. To reappraise the single-factor solution by Klain et al. (2017), the number of factors was fixed to one. The result showed acceptable factor loadings (>0.50) for all seven items (Table 2). The factor showed an eigenvalue of 3.022, and the next eigenvalue was 1.011 (Figure 1).

**TABLE 2:** Result of the principal component analysis with oblique rotation for the seven relational value (RV) items.

		**Factor loading**
RV_IDEN	I have strong feelings about nature (including all plants, animals, the land, etc.); these views are part of who I am and how I live my life.	0.804
RV_HEALTH	My health or the health of my family is related one way or another to the natural environment.	0.698
RV_KIN	Plants and animals, as part of the interdependent web of life, are like “kin” or family to me, so how we treat them matters.	0.682
RV_WILD	I often think of some wild places whose fate I care about and strive to protect, even though I may never see them myself.	0.670
RV_ RESP	How we manage the land, both for plants and animals and for future people, reflects my sense of responsibility to, and so stewardship of the land.	0.644
RV_COMM	There are landscapes that say something about who we are as a community, a people.	0.549
RV_OTHER	Humans have a responsibility to account for our own impacts to the environment because they can harm other people.	0.508
α = 0.775		

To answer whether RVs and CNS are different or overlapping constructs, a third and final PCA with the seven RV and the six CNS items was performed. A two-factor solution was obtained, revealing a partial separation of CNS and RVs. Two CNS items loaded for both factors (Table 3 and Figure 1).

**TABLE 3:** Result of the principal component analysis with oblique rotation for the seven relational value (RV) items and the remaining six Connectedness to Nature Scale (CNS) items.

	**Components**	
	**1**	**2**
CNS_6	0.785	
CNS_5	0.779	
CNS_2	0.766	
CNS_4	0.742	
CNS_1	0.652	
CNS_7	0.628	
RV_KIN	0.568	0.377
RV_IDEN	0.556	0.543
RV_HEALTH		0.672
RV_RESP		0.672
RV_OTHER		0.661
RV_WILD		0.624
RV_COMM		0.540

### Discussion

#### General Considerations

The result of the first factor analysis proves the single-factor solution for our shortened and translated version of CNS. After the lowest loading item (*p* < 0.25) was removed, the loadings of the remaining items increased. Similar results were obtained by Pasca et al. (2017) with a seven-item CNS translated in Spanish. In comparison to this study, we reached a similar Cronbach’s alpha (α = 0.866) and even higher factor loadings than Mayer and Frantz (2004) in the original study (α = 0.84). Therefore, in the case of α, sufficient reliability is proven (Tavakol and Dennick, 2011) that confirms scale usability for our further analysis. In the next step, we examined the coherence of the seven RV items as a single construct for our test group. Klain et al. (2017) previously showed for their test group that six of the seven items cluster together as a single-factor construct. For the test group, the forced single-factor solution has acceptable factor loadings (>0.5) and reliability (α = 0.775).

There are different criteria for determining how many factors need to be maintained in a PCA. One of the most common ones is the Kaiser criterion, which recommends that all factors with an eigenvalue > 1 be preserved (Fabrigar et al., 1999). For the analyzed data set, two factors show an eigenvalue > 1, bringing the number of factors to two. Jolliffe (1972) lowers the cutoff value and suggests extracting factors with an eigenvalue > 0.7, which in our case would mean obtaining three factors. A third commonly used option to determine the number of factors is to consider the point of inflection of the scree plot. The number of factors equals the number of factors on the left side of the inflection point excluding the inflection point itself (Field, 2009). For our data, the scree plot indicates a one-factor solution.

Depending on the criteria used, different numbers of factors are possible from the collected data. The tendency is toward a multi-factor solution. To clarify the RV structure, further analysis (study 3) is required.

#### Relational Value and Connection to Nature

**FIGURE 2:**
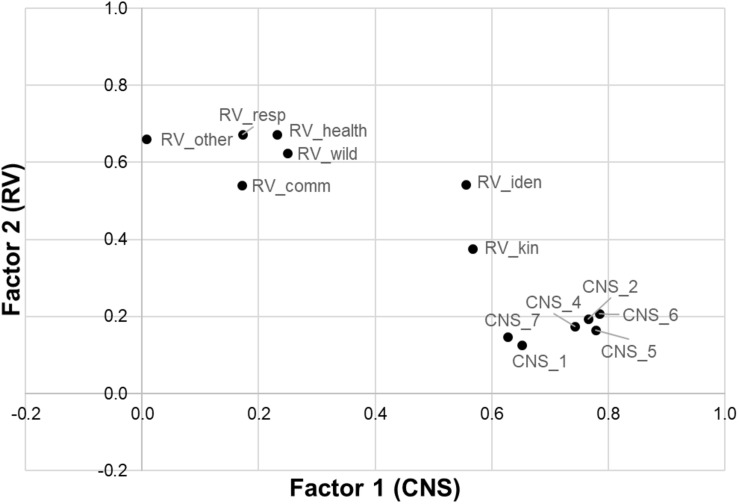
Result of the principal component analysis with oblique rotation for the seven relational value (RV) items and the remaining six Connectedness to Nature Scale (CNS) items. Factor 1 represents CNS, and Factor 2 represents RVs. The results show an overlap between RVs and CNS.

Following these fundamental considerations, we discuss the two main questions of our study. Our results support the hypothesis that RVs and CNS have a certain overlap to some extent. The factor analysis of the CNS and RV items does not show a clear separation into two separate clusters, which would be expected for completely distinct constructs (Figures 1, 2). Two RV items show positive loadings on both factors (>0.3). This outcome could be expected for the following reasons. The first overlapping item, “RV_iden,” consists of a personal question to determine how much a person regards nature as being part of his/her life. The second overlapping item, “RV_kin,” determines whether a person considers nature as some kind of family. Both items directly or indirectly measure some kind of connection to nature, which explains the loadings on both factors. If persons consider nature as being part of their lives or have a familiar solidarity with nature, the connection to nature will be higher. Consequently, CNS cannot be separated as clearly as the NEP from RVs, as revealed by Klain et al. (2017). Therefore, it is reasonable to assume that the concept of RVs includes, to a certain extent, a connection to nature.

## Study 3

After study 2 could not provide sufficient evidence about the factor structure of RVs, we used CFAs on a different sample of students and pupils for further examination. Three models were developed and compared to evaluate the structure of RVs. Additionally, one model was tested to determine the result from study 2 that RVs and connection to nature are overlapping constructs.

### Methods

Based on our findings from study 2, we tested the fit for four different models. Model 1 is a single-factor model with the seven RV items. This model matches with the assumption by Klain et al. (2017) and our forced factor analysis from study 2 (Table 2) that RVs are a single dimensional construct. The second model is a three-factor model based on theoretical assumptions about the concept of RVs and the results from study 2. The items RV_iden and RV_kin that showed affiliation to connection to nature in study 2 form one component (connection). The second component is formed by the items RV_health and RV_comm because both items represent the meaning of the land and environment for the community to some extent. The third component consists of the remaining three items, RV_wild, RV_resp, and RV_other. These items reflect the responsibility for the land.

**FIGURE 3:**
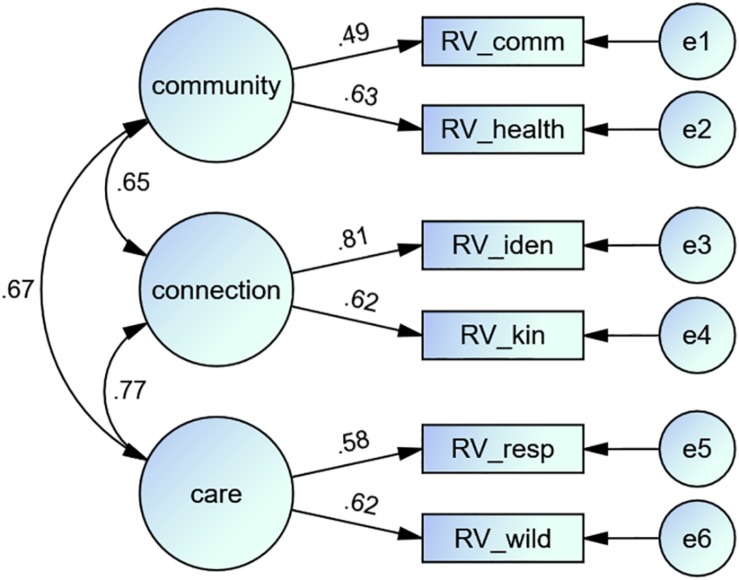
Path diagram showing the result of the confirmatory factor analysis of model 3.

Model 3 is an improved version of Model 2, based on the results of study 2. To enhance model fit, the lowest loading item (RV_other) was removed, bringing the number of RV items to six (Figure 3).

**FIGURE 4:**
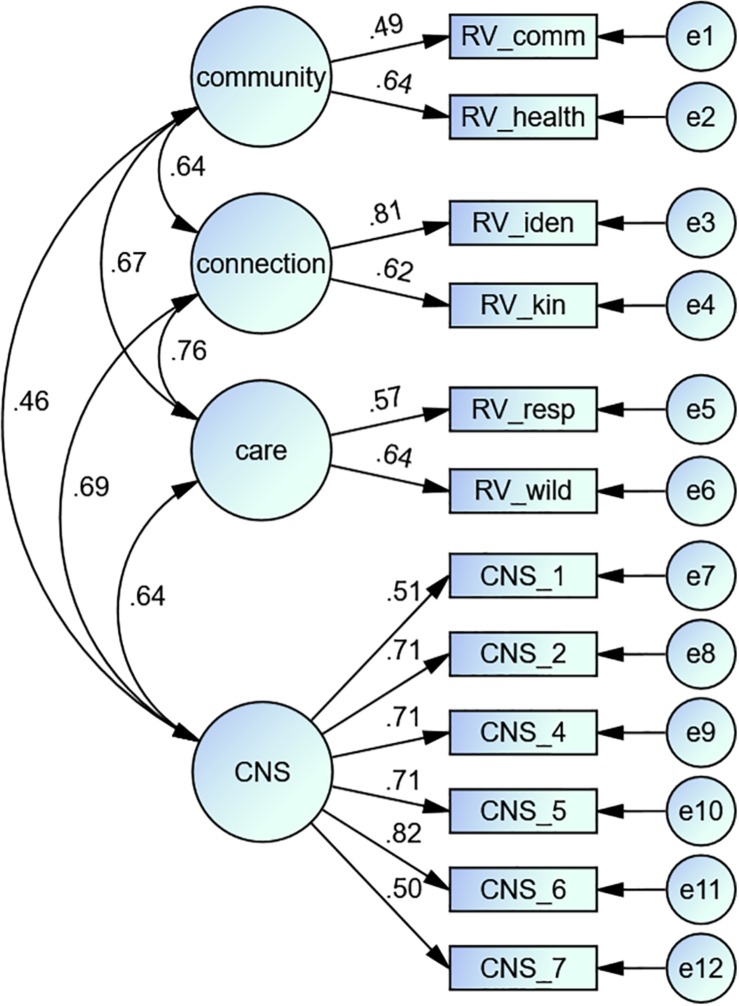
Path diagram showing the result of the confirmatory factor analysis of model 4.

Model 4 was applied to verify the structure of RVs compared to the CNS. The six CNS items form one factor and the six RV items form three factors as in model 3 (Figure 4).

#### Participants

The sample consisted of 878 participants (62.9% female, 36.2% male, 0.3% no answer) and was determined by our goal to reach a meaningful sample size of more than twice the amount as in study 2. Slightly more than half of the participants (*n* = 466) were students from the Goethe-University in Frankfurt (Germany). The majority of university students (>77%) were between 18 and 23 years old. Most of the students were bachelor of science students in biology or other natural sciences (*n* = 261), while the remaining 192 participants were biology teacher trainees. Only 13 participants did not answer the question of which degree they would like to obtain. The survey method was the same as that used in study 1, but half a year later with different students. The remaining 417 participants were pupils (seniors) from local schools. Over 92% of the test persons were aged 15–18 years. In return for the participation in our study, the high school students received free entrance and a guided tour at the Opel Zoo in Kronberg (Germany). The study was advertised by e-mail to the local schools. If individual students did not take part in the survey, they still received free entrance and the guided tour. The survey participation was voluntary, and participants under the legal age were asked for a signed letter of agreement by their parents. Privacy policy has been respected. The data were obtained between October and February 2019 during the winter semester.

#### Analysis

The PCAs were performed using AMOS 26. Missing values in the data set were replaced with series means and as fitting function, maximum likelihood (ML) was used. Frequently used fitting variables to verify the suitability of the model were extracted [χ^2^/df, root mean square error of approximation (RMSEA), standardized root mean square residual (SRMR), Comparative Fit Index (CFI), and Tucker–Lewis Index (TLI)]. For the CFI and TLI values, >0.95 are requested, for RMSEA values, <0.06, and for SRMR, <0.08 (Hu and Bentler, 1999). For a reasonable model fit, the ratio χ^2^/df should be lower than 5 (Wheaton et al., 1977). Carmines and McIver (1981) even suggest three or less.

### Results

Table 4 displays the model fix variables from the four tested models. Figures 3, 4 show the path diagram of the CFAs of models 3 and 4.

**TABLE 4:** Results from confirmatory factor analysis for models 1–4 with a sample of 878 participants.

	**χ^2^**	***p***	**df**	**χ^2^/df**	**RMSEA**	**(S)RMR**	**CFI**	**TLI**
Model 1	156.14	<0.001	14	11.15	0.107	***0.063***	0.868	0.802
Model 2	60.10	<0.001	11	5.463	0.071	***0.044***	***0.954***	0.913
Model 3	12.43	0.053	6	***2.07***	***0.035***	***0.024***	***0.993***	***0.982***
Model 4	152.76	<0.001	48	***3.18***	***0.050***	***0.038***	***0.965***	***0.952***

*Values in the optimal range are printed in italics and bold. χ^2^, chi-square; df, degrees of freedom; RMSEA, root mean square error of approximation; (S)RMR, (standardized) root mean square residual; CFI, Comparative Fit Index; TLI, Tucker–Lewis Index.*

### Discussion

Models 1, 2, and 3 were created to evaluate the structure of RVs, and our results provide evidence that RVs are not a single-factor construct as assumed by our first factor analysis. Model 1 (the single-factor solution) revealed insufficient model fitting with four from five tested fit indicators outside of the optimal range. On this basis, it can be assumed that RVs are a more complex construct, and a single dimension is not the optimal solution for RVs.

The second model includes the theoretical specifications of RVs that are described by Chan et al. (2016) and the results of our analysis in study 2 creating a three-factor model. RVs are not only the personal relation to nature (connectedness to nature) and relationships between people involving nature (community) but also the personal and societal values concerning nature (care). This result coincides with the view of Britto dos Santos and Gould (2018) who also see connectedness, community, and care as important parts of RVs. Model 2 showed a better model fit compared to model 1. Nevertheless, only two of the five fitting indicators were in the optimal range. In contrast to the two previous models, Model 3 has an optimal model fit with all tested fitting variables. Additionally, Model 3 is the only tested model that showed a p-value larger than 0.05 for the chi-square test, adding further evidence that our model fits the data (Schermelleh-Engel et al., 2003). The results provide evidence that RVs are not a single-factor construct as expected but have at least three dimensions.

To examine the relationship between the CNS and RVs, we analyzed a fourth model. Model 4 divided the RV items on the three factors (connectedness, community, and care) and the six CNS items on another one (Figure 4). The CNS showed a high correlation to the connection and care components of RVs (r_connection_ = 0.69; r_care_ = 0.64). This result could be expected because these two components show similarities to the connectedness to nature concept by Schultz (2002). The connection component of RVs coincides with Schultz cognitive component, while the care component is similar to the affective component of nature connectedness. This result is not surprising. Various authors have shown that the concept of connection to nature can be interpreted more broadly and is closely related to concepts such as values or environmental identity. Tam (2013); Olivos and Aragonés (2011), and Brügger et al. (2011) found high correlations between the CNS and other established environmental scales such as the INS Scale and the EID Scale. The EID Scale attempts to capture the relationship between a person’s identity and nature. This includes the connection to nature and natural objects as well as to geographical locations, which is called place identity (Clayton, 2003). This connection to places is also an essential part of the RVs. This example shows how closely related the various concepts of connectedness to nature are to RVs.

These findings show that RVs overlap with the concept of connection to nature. Both concepts contain the relatedness with nature and the environment and the care for natural things. Nevertheless, there are also some differences. RVs have a community component that tries to explain what the land, nature, or the environment means for a group of people. This component is not part of the nature connectedness concept.

The overall analysis provides evidence that RVs and connection to nature are not distinct constructs. RVs seem to contain a substantial part of the connectedness to nature concept but also add some additional content that is not part of connection to nature. For this reason, we assume that RVs are a useful concept to explain people decisions concerning nature and to explain proenvironmental behavior.

## General Discussion

The concept of RVs that emerged in recent times tries to explain people’s decisions concerning nature (Chan et al., 2016). Despite the large number of publications on the topic in the last few years, there is still a lack of empirical research. An important reason for this is probably the lack of an evaluated measurement tool. Therefore, we have examined an existing measurement tool. Using different samples, we provided evidence for convergent and discriminant validity of an RV measurement tool developed and used by Klain et al. (2017). Besides the different kinds of validity, we also prove reliability of the measurement tool by testing test–retest reliability and calculating Cronbach’s alpha. For further analysis of the structure of RVs, we used a series of PCAs as well as CFAs. Since the PCA with the forced single-factor solution of the seven RV items did not show perfect results, the measurement tool was further analyzed using a larger sample size and a series of CFAs. Only the CFA with a three-factor solution for the RV items had sufficient model fit. Therefore, we conclude that RVs are a multidimensional rather than a one-dimensional construct. A comparison of RVs with the well-known concept of nature connectedness by using a shortened version of the CNS by Mayer and Frantz (2004) displayed a high accordance with two RV components. Despite the overlap of the two concepts, RVs are covering social aspects that are not part of nature connectedness. Our empirical study confirms the multidimensionality of the RV concept as assumed in theoretical research. Both Klain et al. (2017) and Britto dos Santos and Gould (2018) consider care and community to be part of the RVs. With the research conducted, this assumption can be empirically validated. In addition, Britto dos Santos and Gould (2018) consider connectedness to nature to be an elementary component of the RVs, which is confirmed by the overlap of RVs and CNS in a PCA in study 2 and CFAs in study 3. RVs are a possible influencing factor on the decisions of economic and political decision makers (Stenseke, 2018), but at the same time, there is a lack of empirical research (Schultz and Martin-Ortega, 2018). A concrete measurement tool for quantifying RVs was needed and is now available.

The multidimensional character of the concept, empirically proven in this study, can be used to justify decisions affecting nature and people, in addition to the intrinsic and instrumental values. If, for example, the care component is particularly developed in a community, it is necessary to pay special attention to it in economic and political decisions. As RVs play a more important role in local communities, decisions at this level should consider them.

The multidimensionality also makes it possible to place the RV construct in concrete relationship with already existing concepts of psychology and environmental psychology. The proof of the care component of RVs shows the concrete link with Stern and Dietz (1994) concept of biospheric values, which refer to the protection of the environment. The identified community component reflects the concept of benevolence by Schwartz (2012), and the connection component of the RV is clearly related to the cognitive component of Schultz (2002) concept of connection to nature. In future research, these aspects should be investigated and considered more closely.

Empirical research on RVs is particularly useful for environmental education. In environmental education research, factors such as environmental knowledge (e.g. Braun and Dierkes, 2017; Schmitz and Da Rocha, 2018), environmental attitudes (e.g. Pooley and O’Connor, 2000; Liefländer and Bogner, 2014), or connection to nature (e.g. Kossack and Bogner, 2012; Liefländer et al., 2013) are often reviewed and related to proenvironmental behavior. With the RVs, a new approach to explaining human behavior that affects nature has been added, which can now also be examined empirically. The three-dimensional instrument can be used to examine the success of environmental education programs and to determine whether the RVs are being promoted. The dimensions enable a differentiated approach in the evaluation of educational programs. It can be determined exactly in which context and extent a program contributes to the development of RVs. This allows environmental education programs to focus on a specific area of the RVs and track its success (or failure). Moreover, this seems particularly useful for regional and local environmental education programs. For example, promoting a concrete link to a place or the importance of that place to the regional community is an essential contribution that goes beyond connection to nature. Creating awareness of nature and place could therefore also be a starting point for future (especially regional) environmental education programs. The RVs have the potential to become a fundamental construct for evaluating the success of environmental education programs, alongside connection to nature, environmental attitudes, environmental values, and environmental knowledge. Especially regional environmental education programs could support local environmental protection by creating a connection between the community and the country or nature.

For this reason, we recommend further research on the topic of RVs especially to figure out the dependency of people’s RVs and proenvironmental behavior. In this respect, the measurement scale of Klain et al. (2017) appears to be an adequate tool, and it can be assumed that there is a positive correlation between RVs and proenvironmental behavior. In this way, RVs could be a new approach to explain people’s environmental behavior, as it is the case with the connection to nature (Kals et al., 1999; Frantz et al., 2005; Kaiser et al., 2008).

## Limitations

Although the study was conducted with great care, some limitations need to be addressed. One methodological limitation is the survey group. University and high school students with a scientific focus were questioned. The respondents’ views are therefore strongly influenced by natural sciences. It is conceivable and possible that students of the humanities, languages, economics, and social sciences would evaluate RVs differently. Furthermore, the selection of this sampling group tended to include people who are more in touch with education. In further research of RVs, it is essential to also include people who are less well educated. In addition, the majority of the respondents were young adults between the ages of 16 and 30. As a result, our study does not reflect the age structure in our western society. It can be assumed that there is a clear value difference between the generations (Inglehart, 1977), which should also be reflected in the RVs. In order to obtain a more precise picture of the construct of the RVs, it will be necessary to conduct further studies with a wider survey group. Moreover, it would be advantageous to cover different cultural areas (compare Schwartz, 1992; Inglehart, 1997).

Another methodological limitation is the use of a shortened Connectedness to Nature Scale. Since the questionnaire was answered during regular courses at the university, it was necessary to keep it as short as possible. Therefore, of the original 14 CNS items, only the six with the highest factor loadings were used. Although the reliability, validity, and correct measurement of reduced CNS were proven (Pasca et al., 2017), it is possible that information related to the RVs was lost due to the reduction. For example, it is conceivable that the overlap of the CNS with RVs would be greater or smaller on a full CNS than it was found in our study. For further research, it is necessary to also determine the relationship between RVs and CNS for the full CNS. In the same way, it would be useful to examine the relationship between RVs and NEP, as considered by Klain et al. (2017), for the complete NEP.

This investigation of the construct is only the beginning to the empirical investigation of the structure of RVs. Further empirical research is required, as well as more theoretical framework to add evidence on the three-factor solution of RVs. Despite our contribution, the demand of Schultz and Martin-Ortega (2018) for more quantitative research on RVs remains.

## Data Availability Statement

The raw data supporting the conclusions of this article will be made available by the authors, without undue reservation, to any qualified researcher.

## Ethics Statement

Ethical review and approval was not required for the study on human participants in accordance with the local legislation and institutional requirements. Written informed consent to participate in this study was provided by the participants’ legal guardian/next of kin.

## Author Contributions

MK and PD conceived and designed the study and contributed to the final version of the manuscript. MK collected the data and performed the analysis and wrote the original draft.

## Conflict of Interest

The authors declare that the research was conducted in the absence of any commercial or financial relationships that could be construed as a potential conflict of interest.

## Appendix 1

**Table:**

RV_IDEN	I have strong feelings about nature (including all plants, animals, the land, etc.) these views are part of who I am and how I live my life.
RV_HEALTH	My health or the health of my family is related one way or another to the natural environment.
RV_KIN	Plants and animals, as part of the interdependent web of life, are like “kin” or family to me, so how we treat them matters.
RV_WILD	I often think of some wild places whose fate I care about and strive to protect, even though I may never see them myself.
RV_ RESP	How ***we*** manage the land, both for plants and animals and for future people, reflects my sense of responsibility to and so stewardship of the land.
RV_COMM	There are landscapes that say something about who we are as a community, a people.
RV_OTHER	Humans have a responsibility to account for our own impacts to the environment because they can harm other people.

**Table:**

RV_IDEN	Ich habe starke Gefühle für die Natur (einschließlich aller Pflanzen, Tieren, dem Land, usw.). Diese Ansichten sind Teil davon, wer ich bin und wie ich mein Leben lebe.
RV_HEALTH	Meine Gesundheit oder die Gesundheit meiner Familie ist auf dem einen oder anderen Weg mit der natürlichen Umgebung verbunden.
RV_KIN	Pflanzen und Tiere, als Teile des verflochtenen Netzwerks des Lebens, sind wie Verwandte oder eine Familie für mich, daher ist es wichtig, wie wir sie behandeln.
RV_WILD	Ich denke oft an unberührte Orte, deren Schicksal mir wichtig ist und die ich schützen möchte, auch wenn ich sie vielleicht nie selbst besuchen werde.
RV_ RESP	Wie wir das Land bewirtschaften, sowohl für Pflanzen als auch für Tiere und zukünftige Menschen, spiegelt unsere Pflicht und Verantwortung für das Land wider.
RV_COMM	Es gibt Landschaften, die etwas darüber aussagen, wer wir als Gemeinschaft, als ein Volk sind.
RV_OTHER	Die Menschen haben die Verantwortung für Auswirkungen auf die Umwelt zu tragen, weil sie anderen Menschen schaden können.
